# Interpersonal communication regarding pregnancy-related services: friends versus health professionals as conduits for information

**DOI:** 10.1186/s12884-018-1729-x

**Published:** 2018-04-12

**Authors:** Leanne Dougherty, Emily Stammer, Thomas W. Valente

**Affiliations:** 1 John Snow, Inc, Rosslyn, VA USA; 2 University of Southern California, Los Angeles, CA USA

**Keywords:** Social network, Maternal health, Pregnancy, Ghana, Health communication

## Abstract

**Background:**

Social network characteristics influence a wide range of health behaviors but few studies examine the relationship between social network characteristics and pregnancy-related outcomes.

**Methods:**

Using a baseline survey from a behavior change pilot project in the Upper West region of Ghana, we examine four outcomes: (1) early antenatal care, (2) having at least four antenatal care visits, (3) skilled birth attendance, and (4) postpartum care. We collected demographic and network data from 1606 women of reproductive age who had a child in the five years preceding the survey. We estimated associations by regressing the four pregnancy-related outcomes on the demographic and network characteristics.

**Results:**

The results suggest that there is little interpersonal communication about pregnancy-related issues, as 60.2% of respondents reported talking to no one. For those women who did talk to someone, communication with a health professional had the strongest association with accessing services (e.g., Adjusted Odds Ratio [AOR] = 8.02, *p* < 0.01, for having a facility birth). Communicating with friends was also significantly associated with outcomes (AOR = 4.23, *p* < 0.0, for having a facility birth).

**Conclusions:**

This study provides evidence that there was little social communication about pregnancy-related issues in these communities at that time, indicating that an intervention to promote such communication could be successful. In addition, women who reported discussing pregnancy-related issues with friends or a health professional were more likely to access a birth facility and have a skilled birth attendant than those who reported discussing the same topics with their partner.

## Background

Despite improvements in maternal health in Ghana over the last 20 years, maternal mortality remains high, and the country fell short in meeting the 2015 Millenium Development Goal targets. The institutional maternal mortality ratio fell from 216 maternal deaths per 100,000 live births in 1990 to 144 deaths per 100,000 live births in 2014. But, the maternal mortality ratio did not reach the target of 54 deaths per 100,000 live births set for 2015 [1]. High rates of poverty in the rural areas coupled with cultural traditions that foster low levels of women’s empowerment continue to undermine health outcomes, particularly in the Upper West region of Ghana.

Program managers are increasingly interested in interventions that foster peer-to-peer communication to change norms and beliefs around pregnancy-related issues. Programs that improve interpersonal communication have the potential to increase information and foster empowerment needed for women to access the reproductive health services thereby lowering maternal mortality rates. Consequently, this study explores how program managers can leverage associations between women’s social networks and antenatal and postpartum care service access to design more effective interventions.

This study uses baseline data collected for a planned intervention to increase access to antenatal and postpartum care services. Program managers can use findings from the study to identify where to strengthen existing relationships that support behavior adoption as well as where to introduce initiatives that can alter the social networks that surround an individual in the community, thereby creating an enabling environment for individuals to practice healthy behaviors [2].

Diffusion of innovations theory, which proposes that interpersonal communication is associated with behavior change provides the theoretical rationale for this investigation [3, 4]. The theory is further buttressed by social network theory, which proposes that information, persuasion, modeling, support, and other interpersonal mechanisms flow through networks of colleagues, friends, and confidants [5–9]. Broadly speaking, then, the research question we propose to answer in this study is: How do women’s social networks and interpersonal communications about pregnancy-related issues influence their access to reproductive health services?

Social network factors influence a wide variety of behaviors, including tobacco and other substance use [10, 11]; obesity and physical activity behaviors [7–9, 12]; policy diffusion [9]; and many more behaviors. Social network theory and analysis have also been used to study health behaviors in developing countries, most notably, the use of modern methods of contraception [13–18] . A number of studies in developed countries have explored the role of social support for health seeking for seeking mental health services during the perinatal period and found that partners, family and friends can play an important role in health seeking behavior [19–22]. There are also a number of studies that have examined qualitatively the role of social support and access to maternal health care in developing countries and found that family plays an important role in access to care [23]. Few if any studies, however, have investigated whether network characteristics might be associated with the use of maternal health care–seeking behavior in developing-country settings from a quantitative perspective [19–21, 24] .

In order to understand how network characteristics and the communication that occurs within these relationships affect pregnancy-related outcomes, we compare three types of variables: (1) demographic characteristics, (2) network characteristics, and (3) interpersonal communication. We hypothesize that demographic characteristics including age, education, nonreligious affiliation, and parity will be positively associated with pregnancy-related outcomes. Many communities had women’s groups prior to the survey which focused on income generating activities. We predict that communication among women in these groups could result in increased knowledge of how to access health care facilities and therefore control for women’s group participation [25]. We also control for listening to the radio at least once per week. Radio programs in the region often address social issues and also may contribute to knowledge about how to access health care facilities [26].

For network characteristics we hypothesize the following four characteristics to be positively associated with access to services: (1) network size, because talking to more people both within and outside formal group structures provides more opportunities to learn or be encouraged to access services; (2) being female, because women are more likely to discuss intimate matters with other women; (3) education, because communicating with more educated peers provides more information; and (4) experience, because communicating with peers who have a lot of experience with pregnancy should provide more knowledge regarding reproductive health services.

An additional important network characteristic from the network perspective is the type of relationship—whether it is a partner, family member, friend, or health professional. We expect that communication with friends will have the strongest association with access to services. Friends often share similar traits and have more intimate communication which we hypothesize leads to more persuasive and increased communication [27].

For communication, we hypothesize that women who discuss pregnancy care frequently, will access services. Moreover, we expect that women who report receiving information and/or advice will be more likely to access services compared with those who do not report receiving information and/or advice. For one, information shared through communication can help women understand how and when to access antenatal and postpartum care services. Women who want such services but do not know how to access them can turn to their peers for information about how and when to do it. Communication may also allow for persuasion. Women uncertain about how and when to access services may turn to their peers, and these peers may persuade them to access services.

In contrast to the diffusion model, health services research often shows that contact with health professionals is associated with accessing services. Those women who have a health professional in their network gain access to health service advice more readily than those who do not name a health care professional. Moreover, women who have accessed such services in the past would be more likely to have health care professionals in their network. Thus, naming a health care professional with access to services is a function of having accessed such services in the past.

Using data from the baseline survey, we explore whether women who access birth facilities and receive antenatal and postpartum care services are more likely to communicate with their network partners about pregnancy and childbirth. Specifically, we compare communication between mothers and their social networks of family and friends with interpersonal communication with a health professional. Further, we investigate if pregnancy-related outcomes are more likely to improve if communication contains information or advice.

## Methods

### Setting and study design

Program staff identified nine potential communities in the three districts for the intervention through consultations with government officials and community leaders during visits to Ghana in the first quarter of 2013. Formative research determined that the communities identified in the Jirapa, Lambussie, and Wa West districts of the Upper West region were similar in sociodemographic composition.

### Sample

We based the sample size calculation on the prevalence of the rarest outcome (i.e., proportion of mothers receiving postpartum care within 48 h after birth), which was 15% at baseline and considered to possibly increase to 20% at endline with 90% power at the 95% significance level. We also estimated that the design effect due to clustering of events within Community-based Health Planning and Services zones and households to be 1.23 and that the nonresponse rate would be 10%. Based on these assumptions and using Stata 11.2 statistical software, we estimated the required sample size for the study to be 1746. This translates to about 582 respondents from each arm of the project for the baseline and the endline surveys. Population estimates from the Ghana Health Service indicated that the actual target population of women between the ages of 15 and 49 was close to the desired sample size. As a result, the study team opted to conduct a census by interviewing all women between the ages of 15 and 49 who had given birth to at least one child in the five years preceding the survey in the nine communities in the three districts.

### Data collection

The local research partner, Kintampo Health Research Centre recruited, hired and managed 30 data collectors locally, who administered the surveys using mobile phones. The data collectors, all of whom had graduated from secondary school, attended a five-day training workshop in November 2013. The workshop covered the basic operation of the mobile phone (a Samsung Galaxy Ace Plus), operation of the survey software (SurveyCTO), and the content of the household survey. Data collection took place between November 2013 and February 2014, with 99% of the interviews completed by January 10, 2014. The local research partner assigned data collectors to specific communities and asked them to reside there during data collection to familiarize themselves with the health services of the community. Three supervisors oversaw three groups of data collectors.

The study team submitted an application for the survey and received approval from the Ghana Health Service Ethical Review Committee. At the household level, the investigators read the consent form to the respondent for participation in the survey and obtained informed written consent from all study participants. Data collectors informed respondents that their answers would remain anonymous in the data entry and reporting process.

### Variable measurement

Data collection and variable construction for the key outcomes followed global standard questions and sequences employed by the Demographic and Health Surveys. Variable definitions are as follows: the proportion of women age 15–49 who had a live birth in the five years preceding the survey who (1) gave birth at a facility, (2) were assisted by a skilled birth provider at the most recent birth, (3) had four or more antenatal care visits, and (4) received a health checkup in the first 48 h after delivery for the most recent birth.

Demographic characteristics considered in the model included age, education, religion, and parity. We measured age by asking “How old were you at your last birthday?” We measured education by asking “Have you ever attended school?” If so, “What is the highest level of school you attended?” We measured religion by asking “What is your religion?” In preliminary analyses, we discovered that the one category of religion for which respondents varied on outcomes was those who reported no religion, so we treated this variable as dichotomous: any religion versus no religious affiliation. We measured parity by asking “How many live births have you had in your lifetime?”

Network characteristics considered in the model included the network partner’s sex, education, experience, and relationship to the respondent. In addition, for network communication variables, we included whether they discussed pregnancy-related care, how often they discussed it, and whether the person gave the respondent information or advice. To elicit network nominations, we asked “Who have you chatted with about breastfeeding or receiving care before or after pregnancy?” If respondents named fewer than three persons, data collectors prompted with “Can you think of anyone else? How about sitting in on a conversation, even if you yourself didn’t say anything?” Once a name was elicited we asked the respondent to indicate “Is [name of contact] male or female?” We asked “What is the highest level of school [name of contact] attended?” We measured the pregnancy-related care of the network partner by combining whether the person had ever (1) been pregnant, (2) had antenatal care, and (3) had postpartum care.

We assessed the relationship between the respondent and the named person by asking “What is your relationship?” Response options included husband/partner, friend, a series of other family members, and a family planning agent or nurse/doctor (classified as a health professional). To measure communication, we asked “Which of the following topics was your conversation with [name of contact] about?” The two options were care received before or after birth and breastfeeding. In this study, the rate of discussing care received was a concern. We then asked how frequently they discussed this topic and finally whether the respondent received information and whether she received advice. In sum, the measures we compare in this study are demographic characteristics of respondents and the persons named; the relationship between the respondent and their network partner; and the type of communication exchanged between the pair.

Finally, in most models we included network size as a control variable and in the dyadic analysis alter rank.

### Analysis plan

We estimated associations by regressing the four pregnancy-related outcomes on the demographic and network characteristics. Given the clustered nature of the data, we estimated random effects models with community as the random effect. Since one community only had 3 respondents (Varimperi), we combined it with another (Olli). In the dyadic model, in which each case is a respondent-alter dyad, we included a second nested level for respondent.

## Results

The response rate of the baseline survey was 92.5%, with 1606 women of the 1736 eligible participating. Of these respondents, some 365 provided one name of a social contact, 201 provided two names, and 80 provided three names. In sum, only 39.8% of respondents indicated they discussed pregnancy-related issues with at least one person. Table 1 compares the full sample with the 639 who named at least one network partner. Two of the outcomes were significantly higher in the network sample compared with the full one: facility birth, 69.2% versus 65.6%, and skilled birth attendant, 67.1% versus 63.1%.

**Table 1:** Frequencies for study variables for the full sample and those who named at least one network partner (the network sample)

	Full Sample (1606)	Network Sample (*N* = 639)	Statistical Significance
Facility birth	65.6%	69.2%	*p* < 0.05
Skilled birth attendant	63.1%	67.1%	*p* < 0.01
4+ antenatal care visits	86.4%	86.2%	NS
Postpartum care within 48 h	70.2%	69.2%	NS
Age categories			
Less than 20	4.6%	5.5%	NS
20–29 years	43.3%	41.6%	
30–39 years	40.1%	40.5%	
40–49 years	12.0%	12.4%	
Education			
No schooling	71.4%	68.1%	NS
Some or all primary	18.4%	19.6%	
Some or all middle	8.84%	10.3%	
Some or all secondary or higher	1.4%	2.0%	
No religion	12.9%	11.4%	NS
Parity			
1 child	19.1%	19.7%	NS
2–3 children	29.2%	30.4%	
4–6 children	38.4%	36.6%	
7 or more children	13.3%	13.3%	
Belong to women’s group	65.4%	67%	NS
Listen to radio at least 1/week	49.9%	49.5%	NS

Over 80% of the respondents were between 20 and 39 years of age, and a majority had no formal schooling. In the network sample, 11.4% report no religious affiliation. Approximately one-third reported having 2 to 3 children, and approximately a third reported having 4 to 6 children. Some 67% belonged to a women’s group, with nearly half reporting listening to the radio at least once per week. In this paper we only analyze data from the 639 respondents who named at least one communication partner, as we are testing for network effects. We detected minimal differences in demographic characteristics of women who named at least one network partner and those who did not. Among those who provided at least one nomination, the total number also varied between Community-based Health Planning and Services zones (from 1.09 to 2.38 persons named).

We compared regression models between the full sample and the network one to determine if demographic characteristics were differentially associated with the outcomes. The results (not shown) indicated that the associations between demographics and outcomes were similar with some notable exceptions: We found that women who participated in women’s groups were more likely to have a facility birth and access postpartum care in the network sample and not the full one. We also found that the number of people a women spoke with about pregnancy and childbirth (i.e. network size) was associated with whether she had a facility birth and skilled birth attendant in the full sample and not the network one.

A majority of network nominees, 62%, were female, with 55% having no education or only primary and 44.6% some higher education. The nominee’s relationship to the respondent was partner, 25%; family member, 15%; friend, 21%; and health professional, 38%. Table 2 compares the network nominees on their characteristics by the rank order of their nomination (i.e., first, second, or third). Later nominees were more likely to be female than earlier ones (1.35 versus 0.58, *p* < 0.01, respectively); had more pregnancy experience than earlier ones (73.7% versus 61.8%, *p* < 0.05, respectively); were more likely to be family members and less likely to be partners than earlier ones; and discussed pregnancy-related issues less frequently than earlier ones. There is some evidence that earlier nominees discussed care but provided less advice than later ones.

**Table 2:** Comparison of network characteristics on sex, education, experience, relationship type, and communication

	Alter Rank			Statistical Significance		
	First	Second	Third	1 vs 2	1 vs 3	2 vs 3
Female	61.8%	67.5%	73.7%	NS	*p* < 0.05	NS
Education						
None	35.8%	47.9%	55.7%	*p* < 0.05	*p* < 0.01	
Primary	5.6%	9.4%	11.4%			
Middle	22.9%	7.0%	8.6%			
Secondary	2.5%	4.2%	1.4%			
Higher	33.2	31.5%	22.9%			
Experience-average (SD)	0.85 (1.30)	1.03 (1.37)	1.35 (1.48)	NS	*p* < 0.01	NS
Relationship						
Partner	25.7%	21.1%	12.8%	*P* < 0.01	*P* < 0.01	*P* < 0.01
Family	21.1%	31.8%	50.0%	*P* < 0.01	*P* < 0.01	*P* < 0.01
Friend	12.8%	12.5%	15.0%	NS	NS	NS
Health professional	38.2%	36.4%	27.5%	NS	NS	NS
Communication						
Discuss care	91.4%	86.1%	85.0%	*p* < 0.05	NS	NS
Discuss-average (SD)	2.72	2.61%	2.16	*P* < 0.01	*P* < 0.01	*P* < 0.01
Gave me information	79.5%	75.7%	71.3%	NS	NS	NS
Gave me advice	31.1%	40.7%	38.7%	*P* < 0.01	NS	NS

Table 3 reports the odds ratios based on logistic regressions of demographic and network characteristics and the four pregnancy-related outcomes. Having no religious affiliation was negatively associated with three of the outcomes (facility birth, skilled birth attendant, and having four or more antenatal care visits). Parity was negatively associated with facility birth (AOR = 0.73, *p* < 0.05). Listening to the radio at least once per week was associated with having a skilled birth attendant (AOR = 1.53, *p* < 0.05) and receiving postpartum care within 48 h (AOR = 1.86, *p* < 0.01).

**Table 3:** Random effects logistic regression (odds ratios) of antenatal and postpartum care outcomes on demographic and network variables (*N* = 639)

	Facility Birth	Skilled Birth Attendant	4+ Antenatal Care Visits	Postpartum Care Within 48 Hrs
Age	0.92	0.87	1.23	1.07
Education	1.35	1.22	1.00	0.88
Nonreligious	0.43**	0.42**	0.46*	0.82
Parity	0.73*	0.79	1.14	0.83
Women’s group	1.52	1.38	0.73	1.16
Radio 1/week	1.44	1.53*	1.54	1.86**
Networks				
Size	1.12	1.15	0.95	1.06
Female	0.45*	0.31**	0.86	0.79
Education	0.76**	0.78*	0.92	0.86
Experience	1.19	1.36**	1.22	1.08
Partner (reference)				
Family	1.18	1.61	0.70	1.41
Friend	4.23**	3.89**	0.72	1.53
Health professional	8.02**	8.08**	1.17	5.40**
Communication				
Discuss care	0.18**	0.21**	1.34	0.65
Frequency discuss	1.36*	1.23	0.74*	1.14
Gave me information	0.76	0.78	1.06	0.81
Gave me advice	0.46**	0.43**	2.30**	1.00

*p < 0.05; **p < 0.01

For the network variables, naming female and educated network partners was negatively associated with facility birth (AOR = 0.45, *p* < 0.05; AOR = 0.76, *p* < 0.01, respectively) and having a skilled birth attendant (AOR = 0.31, *p* < 0.01; AOR = 0.78, *p* < 0.05, respectively). Naming more experienced partners was positively associated with having a skilled birth attendant (AOR = 1.36, *p* < 0.01). Naming friends and naming a health professional were positively associated with facility birth (AOR = 4.23, *p* < 0.01; AOR = 8.02, *p* < 0.01, respectively) and having a skilled birth attendant (AOR = 3.89, *p* < 0.01; AOR = 8.08, *p* < 0.0, respectively), and naming a health professional was associated with obtaining postpartum care within 48 h (AOR = 5.40, *p* < 0.0).

In terms of communication, discussing pregnancy-related care was negatively associated with facility birth (AOR = 0.18, *p* < 0.01) and having a skilled birth attendant (AOR = 0.21, *p* < 0.01). The frequency of discussion was positively associated with facility birth (AOR = 1.36., *p* < 0.05) but negatively associated with four or more antenatal care visits (AOR = 0.74, *p* < 0.05). Reporting that the network partner provided advice was negatively associated with facility birth and skilled birth attendant (AOR = 0.46, *p* < 0.01; AOR = 0.43, *p* < 0.01, respectively) but positively associated with receiving four or more antenatal care visits (AOR = 2.30, *p* < 0.05).

The variables associated with skilled birth attendance were listening to the radio at least once per week (AOR = 1.78, *p* < 0.01); nominating a friend (AOR = 3.43, *p* < 0.05); and nominating a health professional (AOR = 11.71, *p* < 0.001). Women who identified themselves as traditional/spiritualist or did not practice religion were less likely to access a skilled birth attendant (AOR = 0.52, *p* < 0.05) as were women who had more than 1 child and living in the same district when compared with living in the same household/compound. Listening to the radio at least once per week was also associated with receiving postpartum care in the first 48 h after delivery (AOR = 2.00, *p* < 0.01).

Table 4 repeats the regression analyses on the dyadic data so that each case is a respondent-alter. This enables the analyses to include all nominations (not just the first one). We included a control variable for alter rank but did not include this in the results because we did not find a relationship with any of the outcomes. These results are similar to those reported using only the first named alter, with the exception that some variables either attained or lost their statistical significance. Specifically, for facility birth, respondent education, belonging to a women’s group, and listening to the radio once per week attained statistical significance; female network partners, discussion frequency, and getting advice lost their statistical significance. For skilled birth attendant, belonging to a women’s group attained statistical significance, and experienced network partners lost it. For four or more antenatal care visits, listening to the radio once per week became statistically significant.

**Table 4:** Logistic regression (odds ratios) antenatal and postpartum care outcomes on demographic and network variables with the data reshaped to be dyadic (*N* = 999)

	Facility Birth	Skilled Birth Attendant	4+ Antenatal Care Visits	Postpartum Care Within 48 Hrs
Age	0.94	0.86	1.2	1.13
Education	1.44**	1.26	0.99	0.83
Nonreligious	0.48**	0.42**	0.41*	0.7
Parity	0.76*	0.8	1.17	0.84
Women’s group	1.70*	1.63**	0.67	1.16
Radio 1/week	1.60*	1.67**	1.52*	1.90**
Networks				
Size	1.06	1.1	1.03	1.07
Alter rank	0.98	0.97	1.01	0.95
Female	0.70	0.56*	0.95	0.85
Education	0.82*	0.85*	1.01	0.95
Experience	1.07	1.18	1.09	1.09
Partner (reference)				
Family	1.03	1.45	0.89	1.01
Friend	2.56*	2.50*	0.67	1.1
Health professional	2.92**	2.97**	0.88	2.07*
Communication				
Discuss care	0.53*	0.53*	0.92	0.67
Frequency discuss	1.08	1.0	0.79*	1.02
Gave me information	0.69	0.65	1.15	0.95
Gave me advice	0.60	0.50**	2.08**	0.94

*p < 0.05; **p < 0.01

## Discussion

The main finding reported in this paper is that women who reported discussing pregnancy-related issues with friends or a health professional were more likely to have accessed a birth facility and had a skilled birth attendant than those who reported discussing them with their partner. In addition, those who reported discussions with a health professional were more likely to have accessed postpartum care within 48 h of their most recent birth. The magnitude of the health professional association was more than twice that of the friend one. Thus, as of November–December 2013, partner and family communication were not associated with access to pregnancy-related services, whereas friend and health professional communication were. The association with health professional communication may be a product of having accessed services in the past, which created an interpersonal connection between the mother and health care professional. In other words, the association is likely a reflection of the influence occurring in both directions: health professional communication led to accessing services and accessing services led to health professional communication. This finding indicates that there existed at this time a substantial barrier to accessing pregnancy-related care, as it required prior access or communication with a health professional, of which there are few in the region.

A second significant finding was that access to facility birth and use of a skilled birth attendant was associated with communication with males and those having less education. There are two implications here: One is that in this region at that time women were dependent on men for information and access to pregnancy-related facilities, thus again establishing a significant barrier to care. The second is that access to services was associated with communication with network partners with less education. Thus, potentially, the information women receive from their networks may be less accurate than communications with those with higher levels of education.

A third significant finding was that women who reported discussing pregnancy-related care and those who reported receiving advice were less likely to have accessed a birth facility or a skilled birth attendant. At the same time, those who reported more frequent communication with the network partner had a higher likelihood of accessing such services. This probably indicates that women are having general discussions about accessing services and not specific detailed information exchanges. The conversations are in all likelihood casual. Conversely, infrequent conversations in which women receive advice were associated with receiving four or more antenatal care visits. If advice women receive particularly from friends and family does not support use of pregnancy related services, this may explain why less advice is associated with more antenatal care visits.

Although both friend and health professional communications were associated with accessing care, the magnitude for the health professional association was much higher, as previously noted. Women were much more likely (three times as likely, 38.2% versus 12.8% for the first person named) to report communication with a health professional (see Table 2). This further underscores the first point above that the interpersonal communication in this region and at this time promoting access to services was largely a product of communication with a health professional. A woman’s pregnancy status may also influence whether or not she cites communication with health providers. For example, women who have a problem related to their pregnancy or their gestational month may predict when she speaks with a health provider.

Finally, women reported very few communication partners—60.2% reported talking to no one. The low number of communication partners was surprising and is not consistent with other egocentric network research (10,13). The low number of communication partners could be because respondents did not discuss pregnancy-related issues with their peers. Exploratory interviews conducted in the selected intervention communities prior to the baseline found evidence of cultural rituals that encouraged women to hide their pregnancies until other woman announced the pregnancy to the community by throwing water on the woman when she emerged from her house. Strong cultural traditions and beliefs in evil and supernatural influences related to pregnancy are widespread in Africa and encourage women to conceal their pregnancies [28]. These cultural traditions could explain why we found fewer numbers of network partners. Another possible explanation may be due to interviewer bias, which we documented in a separate study [29]. The study concluded that the decrease in network size could be in part a function of interviewers learning that collecting no or few names shortened the length of the interview and in part from diffusion of this information among the interviewers. We documented a negative association between network size and time of interview, so that respondents interviewed later in the data collection process reported fewer communication partners. Thus, there was variability between communities in whether any and how many names were provided in response to the network question. Oddly enough, there was a very weak negative correlation between network size and parity (*r* = − 0.05, *p* = 0.06). Taken with the findings above regarding communication with health professionals, it is clear that there is little interpersonal communication around pregnancy- related issues.

Clearly our research hypotheses were not supported in this setting, indicating that there is very little diffusion of information or advice to access pregnancy-related services in these communities at that time. The lack of diffusion of such information has created a climate in which services are accessed less frequently than they should be, and this results in higher rates of maternal mortality than desired.

## Conclusion

In sum, these data indicate women in these districts of northwest Ghana have not prioritized communication among themselves and certainly not with their partners or family members about pregnancy-related issues. Health professional communication seems the only route in which women would have learned about such services, and this association is likely a consequence of having received services prior to such communications. Given that over 60% of women participated in a woman’s group, program managers may want to consider prioritizing communication interventions to this specific target population. This study is a baseline assessment of these communications for an intervention designed to promote reproductive health services through interpersonal communication networks. The baseline assessment reported here indicates that there is considerable need for such communications to increase. However, future research should focus on understanding women’s intercommunication network preferences about pregnancy and childbirth and who they would trust to communicate with and why.
